# Ocean Warming and CO_2_-Induced Acidification Impact the Lipid Content of a Marine Predatory Gastropod

**DOI:** 10.3390/md13106019

**Published:** 2015-09-24

**Authors:** Roselyn Valles-Regino, Rick Tate, Brendan Kelaher, Dale Savins, Ashley Dowell, Kirsten Benkendorff

**Affiliations:** 1 Marine Ecology Research Center, School of Environment, Science and Engineering, Southern Cross University, P.O. Box 157, Lismore, NSW 2480, Australia; E-Mail: r.regino.10@student.scu.edu.au; 2 National Marine Science Centre, Southern Cross University, Coffs Harbour, NSW 2450, Australia; E-Mails: ricky.tate22@gmail.com (R.T.); brendan.kelaher@scu.edu.au (B.K.); 3 Southern Cross Plant Science, Southern Cross University, Lismore, NSW 2480, Australia; E-Mails: dale.savins@scu.edu.au (D.S.); ashley.dowell@scu.edu.au (A.D.)

**Keywords:** marine lipids, ocean climate change, *Dicathais orbita*, polyunsaturated fatty acids, *n-*3, *n-*6, plasmalogens

## Abstract

Ocean warming and acidification are current global environmental challenges impacting aquatic organisms. A shift in conditions outside the optimal environmental range for marine species is likely to generate stress that could impact metabolic activity, with consequences for the biosynthesis of marine lipids. The aim of this study was to investigate differences in the lipid content of *Dicathais orbita* exposed to current and predicted future climate change scenarios. The whelks were exposed to a combination of temperature and CO_2_-induced acidification treatments in controlled flowthrough seawater mesocosms for 35 days. Under current conditions, *D. orbita* foot tissue has an average of 6 mg lipid/g tissue, but at predicted future ocean temperatures, the total lipid content dropped significantly, to almost half. The fatty acid composition is dominated by polyunsaturated fatty acids (PUFA 52%) with an *n-*3:6 fatty acid ratio of almost 2, which remains unchanged under future ocean conditions. However, we detected an interactive effect of temperature and *p*CO_2_ on the % PUFAs and *n-*3 and *n-*6 fatty acids were significantly reduced by elevated water temperature, while both the saturated and monounsaturated fatty acids were significantly reduced under increased *p*CO_2_ acidifying conditions. The present study indicates the potential for relatively small predicted changes in ocean conditions to reduce lipid reserves and alter the fatty acid composition of a predatory marine mollusc. This has potential implications for the growth and survivorship of whelks under future conditions, but only minimal implications for human consumption of *D. orbita* as nutritional seafood are predicted.

## 1. Introduction

Climate change is one of the major environmental challenges to humankind and all other life forms on Earth. The consequences of the global climate change have been widely reported [1,2,3] and are likely to worsen over the coming decades. Global sea surface temperatures are projected to increase by 2–4 °C towards the end of the 21st century [1], while the surface ocean pH is predicted to decrease by 0.14–0.35 units, adding to the present decrease of 0.1 units that has already occurred since pre-industrial times [1,2]. The drop in pH is due to the absorption of increasingly greater CO_2_ concentrations into the oceans as a result of the rising partial pressure of atmospheric CO_2_ (*p*CO_2_) from anthropogenic activities, such as burning of fossil fuels, agriculture and land clearing [1]. These two phenomena, ocean warming and *p*CO_2_-induced acidification, are anticipated to have detrimental effects on seawater quality and consequently to marine organisms.

As soft-bodied, slow moving invertebrates, molluscs are highly susceptible to biotic pressures and abiotic changes in their environment. Marine molluscs are considered good models for climate change studies because they are ectothermic animals with limited ability to regulate their internal temperature. They can only survive within a narrow range of tolerable temperatures to which they have adapted [4] and stress outside their optimal temperature range leads to loss of metabolic functions [3,4,5]. Molluscs are also vulnerable to ocean acidification because their calcium carbonate shells are eroded when exposed to low pH [6,7] and an excess of hydrogen ions in the ocean can interfere with shell formation [8]. Furthermore, molluscs tend to have low metabolic rates and cannot easily compensate for pH disturbances [9]. Chronic stress from sub-optimal conditions can result in decreased growth and reproduction, increased susceptibility to disease and reduced survivorship [3,10,11]. Recent meta-analyses have identified molluscs as one of the most vulnerable invertebrate taxa under changing ocean conditions [9,11,12]. However, further studies are required to investigate the biochemical responses that may lead to reduced resilience under future conditions.

Marine molluscs comprise a major invertebrate fishery resource [13,14] and are highly regarded as healthful food for human consumption [15,16,17,18]. Like other shellfish and fish, molluscs are known to contain significant amounts of lipid, that is relatively low in saturated fatty acids (SFA) and high in polyunsaturated fatty acids (PUFA) [19,20]. The large amounts of PUFA in seafood offer nutritional and health benefits, such as the provision of essential fatty acids, carriers of fat-soluble vitamins, and decreasing the risk of cardiovascular disease [21]. Seafood is not only a good source of PUFAs, but also provides *n-*3 fatty acids with optimal *n-*3/*n-*6 ratios [22]. Dietary intake of *n-*3 fatty acids has a broad range of beneficial health effects in humans, including well-established anti-inflammatory, anti-arrhythmic and prothrombotic properties [23]. They are also reported to reduce the risk of colorectal cancer [24], lower blood pressure associated with hypertension [25], reduce depression and treat type 2 diabetes [26], and Alzheimer’s disease [27]. Omega-6 fatty acids are necessary for good nutrition, but when consumed in large amounts they may become harmful to the human body [26] by increasing the risk of cardiovascular and coronary heart disease [28] and may increase offspring adiposity [29]. To our knowledge, no previous studies have investigated the combined impacts of ocean warming and acidification on fatty acid compositions in marine molluscs, despite possible implications for human health.

Previous studies have shown that fatty acids in marine organisms can be influenced by environmental conditions, such as changes in temperature [30] and CO_2_ concentrations [31]. Temperature is a key parameter for optimal physiology in organisms, since it modulates the basic rates of all chemical reactions in cells, thus affecting the stability of structural components, particularly lipids and proteins [32]. Studies on plankton [33,34], copepods [35], microalgae [32,36,37], and bacteria [38] have demonstrated that temperature impacts the lipid composition by increasing the unsaturated fatty acid levels as the temperature declines and increasing the saturated fatty acids (SFA) content as the temperature increases. A recent study on the interactive effects of temperature and food quality was demonstrated in *Daphnia pulex*, where elevated temperature (26 °C) significantly decreased the total body fatty acids and thus negatively affected the PUFA content [39]. Based on comparison of the fatty acid composition of temperate and arctic marine ectotherms, Lewis [40] proposed that fatty acid profiles could actually be used to predict environmental temperatures effects.

Fewer studies have investigated the effects of ocean acidification (OA) on fatty acid composition. However, in a laboratory experiment to test potential OA effects on fatty acid composition in diatoms, elevated CO_2_ significantly changed the fatty acid concentration and composition of *Thalassiosira pseudonana* [31]. A significant decline in total fatty acids and the ratio of long-chain polyunsaturated to saturated fatty acids (PUFA:SFA) was found in algae cultured under elevated (*p*CO_2_ = 750 ppm; pH = 8.14) compared to present day CO_2_ concentrations (*p*CO_2_ = 380 ppm; pH = 7.94). This impact was directly translated to micro-algal grazing copepods, resulting in an almost tenfold decline in total fatty acids and triple the contribution of SFAs in copepods at high CO_2_ [31]. This study demonstrates the potential for far-reaching consequences of OA in ocean food webs by changing the nutritional quality of essential macromolecules in primary producers that can then cascade up the food web. However, to date, few studies appear to have investigated the potential for synergistic effects of elevated temperature and acidification on the lipid composition of benthic marine predators.

The Muricidae are a diverse family of predatory marine gastropods that currently comprise over 39% of the world-wide gastropod fisheries harvest [41]. Muricidae fisheries production has experienced a slow increase globally, with only 5000–24,000 tonnes of catch annually from 2002 to 2006, then peaking in 2007, with a catch of about 27,000 tonnes [41]. China is the lead producer [41], although muricids have gained importance in small-scale fisheries industry and aquaculture throughout Asia, Europe, and Central and South America [13,42], both for seafood consumption and as a source of Tyrian purple [43,44,45]. *Dicathais orbita* (Gmelin) is temperate species of Muricidae native to Australia, and is considered as a useful bioindicator of environmental conditions [45,46,47,48]. *D. orbita* has also provided a good model for natural product research [45]. However, the fatty acid composition of flesh of this species has not previously been reported.

Thus, the aim of the study was to assess the fatty acid composition in the foot tissue of the *D. orbita* and to investigate any impacts of ocean warming and acidification on the lipid content and fatty acid profile after a 35-day exposure to future climate change conditions. This study provides valuable information to establish the nutritional value of this Australian whelk and provides insights into the potential effect of future ocean conditions on the fatty acid composition of a predatory gastropod, with potential flow-on effects for human consumption.

## 2. Results and Discussion

### 2.1. Impacts of Ocean Climate Change on D. orbita Total Lipid Content

**Figure 1:**
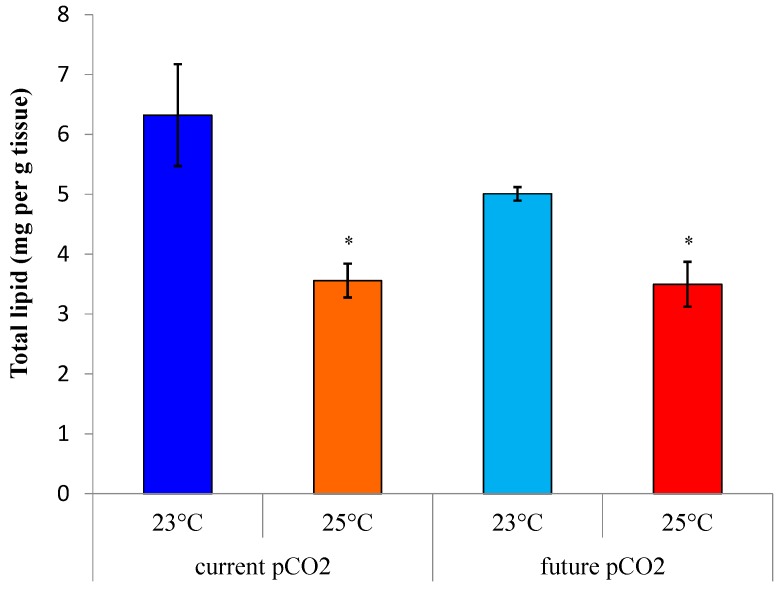
Total lipid yield extracted from the foot tissue of *D. orbita* after 35 days exposure to temperature and *p*CO_2_ treatments (*n* = 6 per group). Error bars show standard error of the mean. * Significantly different to the control at 23 °C and current *p*CO_2_ (*p* < 0.05).

The highest lipid content from *D. orbita* foot tissue was 6.3 mg/g fresh weight in specimens held at current ocean conditions of 23 °C, current *p*CO_2_ (control), dropping to an average of 5.0 mg/g in specimens maintained at 23 °C and future *p*CO_2_ (Figure 1). The lipid content was less than 4% for all whelks kept at 25 °C, with the lowest mean amount of lipid at 3.6 mg/g from the foot tissue of whelks held in future warming and acidification conditions for 35 days (Figure 1). Permutational univariate analysis (PERMANOVA) revealed that temperature (*p* < 0.05), but not CO_2_-induced acidification, significantly affected the total lipid yield and there was no significant interaction between these factors (*p* > 0.05, Table 1). The reduced lipid content under elevated water temperature conditions in this study suggests that *D. orbita* is sensitive to the relatively small increases (~2 °C) in temperature predicted from global climate change models [1]. This result substantiates the negative response to elevated temperature in lipid synthesis for the larvae of the hard clam *Mercenaria mercenaria* and bay scallop *Argopecten irradians* [49], although these larva also showed a reduction in total lipids at elevated CO_2_. Metabolic rates generally increase at higher temperatures and under stressful conditions, thus placing more demand on metabolic reserves, such as the stored lipid. Reduced lipid reserves under conditions of elevated temperature could have significant implications for the long-term viability of whelk populations. High water temperature can result in the dysfunction of important biological functions, as lipids are structural components of all cell membranes and are considered cellular fuels [50,51,52]. Reduced lipid storage has been correlated with poor growth condition in predatory fish in response to increasing sea surface temperatures and it has been argued that lipid reserves also provide a good indicator for reproductive performance, as they are crucial for pre-spawning conditioning of eggs [53]. Consequently, the effects of long-term exposure to future ocean conditions on lipid reserves could result in reduced reproduction, development and growth of marine molluscs. This could have serious implications for population viability and sustainable fisheries, unless they are able to adapt to the new conditions. The east coast of Australia has been recognized as an ocean warming hotspot [54] and in this study, *D. orbita* was collected close to the northern limit of its distribution. Previous studies on invertebrates from thermally stressful intertidal habitats indicate that warm-adapted populations may already be close to their upper thermal tolerance and thus are most vulnerable to ocean warming due to less capacity to acclimatize [55]. Nevertheless, multi-generational studies have indicated the presence of tolerant genotypes in other marine invertebrates [56], suggesting potential for some species to adapt to concurrent ocean warming and acidification.

**Table 1:** Summary of the statistical outcomes for all univariate and multivariate analyses. Two factor PERMANOVAs were used to test the effects of temperature and *p*CO_2_ induced acidification. Significant effects are in bold.

	Temperature		Acidification		Temperature × Acidification	
	Pseudo *F*	*p* Value	Pseudo *F*	*p* Value	Pseudo *F*	*p* Value
UNIVARIATE						
Total lipid yield	19.1230	**0.0002**	1.9863	0.1796	1.6396	0.2098
SFA	3.6189	0.0733	4.2989	**0.0478**	3.1075	0.0905
MUFA	0.4115	0.5451	5.7182	**0.0196**	0.0063	0.9373
PUFA	10.981	**0.0029**	0.0281	0.8705	6.7298	**0.0164**
*n-*3	4.7150	**0.0476**	0.0002	0.9881	1.8572	0.1848
*n-*6	5.1551	**0.0340**	1.2660	0.2742	0.0452	0.8261
*n-*3:*n-*6 ratio	0.33697	0.5697	0.20287	0.6637	1.0828	0.3088
MULTIVARIATE						
Overall fatty acid composition	7.7094	0.0001	2.8452	0.0229	1.7186	0.1321

### 2.2. Impacts of Ocean Climate Change on the Major Classes of Fatty Acids

**Figure 2:**
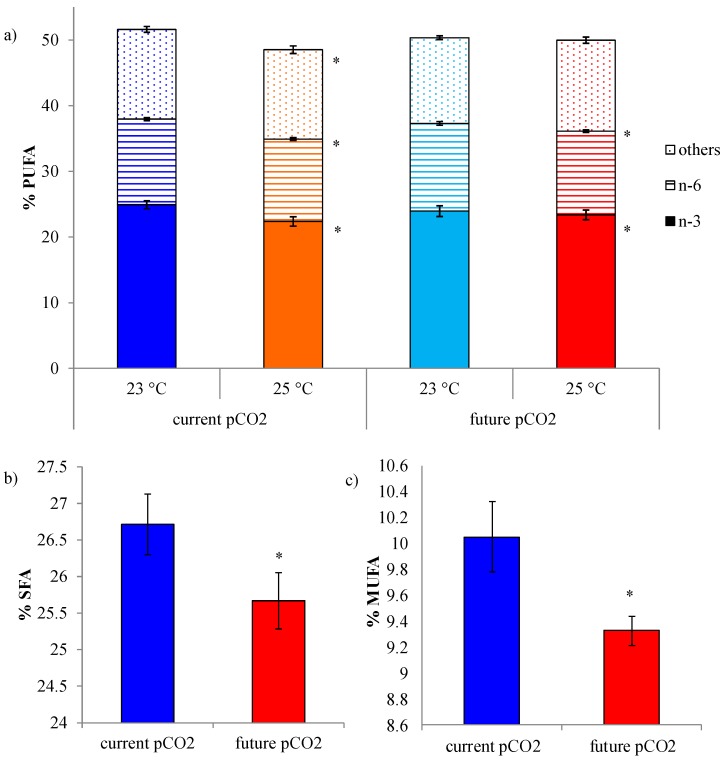
Proportions of (**a**) polysaturated fatty acids (PUFA) (*n* = 6) showing relative amounts of *n-*3 and *n-*6 fatty acids; (**b**) saturated fatty acids (SFA) (*n* = 12) and (**c**) monosaturated fatty acids (MUFA) (*n* = 12) in *D. orbita* foot tissue after 35 days exposure to different temperatures and CO_2_-induced acidification. Error bars show standard error of the mean. * Significant differences (*p* < 0.05) within the same group of fatty acids, in comparison to the control at 23 °C and current *p*CO_2_.

The distribution of fatty acids in *D. orbita* under current ocean conditions was characterised by a predominance of PUFA (51.6% ± 2.1%) followed by SFA (26.7% ± 1.4%) and a low abundance of MUFA (10.2% ± 0.9%) (Figure 2). This relatively high unsaturated fatty acid content is consist across a wide range of molluscs, including filter feeding bivalves, herbivorous gastropods and predatory whelks [20,57]. *D. orbita* contains more *n-*3 (ALA, eicosatrienoic, EPA, DPA, and DHA) than *n-*6 (LA, and ARA) PUFAs (Table 2), with an *n-*3:*n-*6 ratio of approximately 2 (Figure 2). Both *n-*3 and *n-*6 fatty acids are required for normal human health, with a recommended ratio between 1 and 4 [58,59]. In Western diets the *n-*3 to *n-*6 ratio is approximately 1:20–30, indicating a deficiency of *n-*3 fatty acids, which has been linked to several diseases, such as heart disease, diabetes and hypertension [60]. Our study reveals that *D. orbita* provides a good balance of *n-*3 and *n-*6 and a high proportion of PUFAs, which could contribute to a healthful human diet.

Examination of the fatty acids of *D. orbita* after exposure to future temperature and *p*CO_2_-induced acidification treatments for 35 days revealed that the relative proportions of the major fatty acid classes remained similar across all treatments (Figure 2) and there was no significant difference in the ratio of *n-*3:*n-*6 (Table 1, *p* > 0.05). This implies that the nutritional benefit associated with the fatty acid content of these whelks is likely to be retained under future ocean conditions.

**Table 2:** Fatty acid and lipophilic hydrocarbon profile of *D. orbita* foot tissue after 35 days exposure to current and future ocean temperatures and acidification conditions. Data are expressed as % of total fatty acid methyl esters (FAMEs) as mean ± SE (*n* = 12). Others include cyclopropane fatty acids and dimethyl acetal aldehydes from plasmalogen phospholipids.

Fatty Acid	Trivial Name	Retention Time (min)	23 °C, Current *p*CO_2_	23 °C, Future *p*CO_2_	25 °C, Current *p*CO_2_	25 °C, Future *p*CO_2_
Saturated						
C14:0	Myristic	16.8	1.46 ± 0.13	1.56 ± 0.05	1.74 ± 0.08	1.28 ± 0.12
C15:0	Pentadecanoic	18.4	1.24 ± 0.18	1.30 ± 0.07	1.38 ± 0.11	1.11 ± 0.12
C16:0	Palmitic	19.8	9.26 ± 0.49	8.86 ± 0.17	9.53 ± 0.39	8.66 ± 0.23
C17:0	Margaric	21.3	1.98 ± 0.12	1.71 ± 0.10	1.31 ± 0.12	1.22 ± 0.13
C18:0	Stearic	22.6	8.26 ± 0.49	8.17 ± 0.15	7.90 ± 0.24	8.30 ± 0.12
C24:0	Lignoceric	29.7	4.54 ± 0.16	4.99 ± 0.36	4.81 ± 0.16	4.18 ± 0.13
Monounsaturated						
C16:1	Palmitoleic	20.6	1.87 ± 0.16	0.84 ± 0.32	0.89 ± 0.25	0.86 ± 0.23
C18:1 (*n*-9)	Oleic	23.1	4.54 ± 0.49	4.96 ± 0.07	4.89 ± 0.23	4.42 ± 0.06
C20:1 (*n*-9)	11-Eicosenoic	25.7	3.50 ± 0.11	3.40 ± 0.19	3.81 ± 0.16	3.81 ± 0.19
C22:1 (*n*-9)	Erucic	30.2	0.24 ± 0.04	0.20 ± 0.01	0.34 ± 0.11	0.15 ± 0.07
Polyunsaturated						
C18:2 (*n*-6)	Linoleic acid (LA)	24.1	1.54 ± 0.09	1.51 ± 0.07	1.66 ± 0.12	1.69 ± 0.08
C18:3 (*n*-3)	α-Linolenic (ALA)	25.1	0.56 ± 0.04	0.60 ± 0.03	0.69 ± 0.06	0.77 ± 0.04
C20:2	Eicosadienoic	26.5	2.50 ± 0.13	2.89 ± 0.16	2.60 ± 0.1	2.45 ± 0.13
C20:3 (*n*-3)	Eicosatrienoic	27.1	0.03 ± 0.03	0.03 ± 0.03	0	0
C20:4 (*n*-6)	Arachidonic (ARA)	27.3	11.49 ± 0.22	11.84 ± 0.26	10.88 ± 0.16	11.07 ± 0.14
C20:5 (*n*-3)	Eicosapentaenoic (EPA)	28.3	2.61 ± 0.30	2.76 ± 0.33	2.00 ± 0.42	2.05 ± 0.44
C22:2	Docosadienoic	28.5	11.15 ± 0.57	10.15 ± 0.39	11.81 ± 0.51	11.40 ± 0.42
C22:5 (*n*-3)	Docosapentaenoic (DPA)	30.7	17.72 ± 0.49	16.87 ± 0.52	16.13 ± 0.40	16.69 ± 0.39
C22:6 (*n*-3)	Docosahexaenoic (DHA)	30.9	4.02 ± 0.27	3.70 ± 0.13	3.55 ± 0.26	3.85 ± 0.35
Others						
	2-octylcyclo-propanedecanoic	26.8	0.63 ± 0.02	0.64 ± 0.04	0.68 ± 0.03	0.73 ± 0.02
	Unknown fatty acid derivative	29.2	0.53 ± 0.04	0.48 ± 0.06	0.56 ± 0.05	0.50 ± 0.05
Dimethyl acetal aldehydes						
	Hexadecan*-*1-al	18.8	1.04 ± 0.08	0.94 ± 0.04	0.87 ± 0.70	0.82 ± 0.02
	Heptadecan-1-al	20.2	0.21 ± 0.05	1.03 ± 0.26	0.86 ± 0.17	1.78 ± 0.06
	Octadecan-1-al	21.7	8.91 ± 0.83	10.27 ± 0.20	10.80 ± 0.29	11.92 ± 0.20
	Nonadecan-1-al	24.7	0.17 ± 0.06	0.30 ± 0.03	0.30 ± 0.07	0.29 ± 0.06

Nevertheless, PERMANOVA univariate analysis identified some significant effects on the % PUFA, MUFA and SFAs (Table 1). The relative proportion of PUFAs was affected by a significant interaction between temperature and *p*CO_2_ (Table 1, *p* < 0.05). There was a significant reduction in the % PUFAs at elevated temperatures under current *p*CO_2_ conditions, but no effect under future conditions (Figure 2). Temperature also caused a consistent reduction in the amount of *n-*3 and *n-*6 fatty acids, as a percent of total lipids (Figure 2, Table 1, *p* < 0.05). This effect of temperature is consistent with a previous study on bivalves, *Ruditapes decussatus* and *Ruditapes philippinarum*, which also showed decreased PUFA content after exposure to warmer waters [61]. Some PUFAs are important in maintaining the fluidity and permeability of biological membranes in response to temperature fluctuations [50]. The decreased proportion of PUFA at higher temperatures could be explained by acclimatization to the prevailing thermal conditions by restructuring cell membranes to maintain optimal fluidity and permeability. This strategy, termed homeoviscous adaptation, can occur via changes in phospholipid head groups, fatty acid composition, and cholesterol content [51]. Nevertheless, it remains unclear whether this biochemical acclimatization response, to offset the direct effects of temperature on membrane lipid fluidity, can be sustained under long-term conditions that also cause an overall reduction in the total lipid content.

The proportion of PUFAs in *D. orbita* was not significantly affected by future *p*CO_2_ conditions at either temperature (Table 1, *p* > 0.5, Figure 2a). This result agrees with recent research on Pacific oyster *Crassostrea gigas* that showed no effect on the total fatty acid contents under elevated *p*CO_2_ [62]. Nevertheless, exposure to CO_2_-induced acidification did result in a significant but small reduction (1%–2%) in the relative proportions of SFA and MUFA in *D. orbita* (Table 1, *p* < 0.05, Figure 2). Although response to CO_2_ acidification is species-specific, it has been proposed that elevated CO_2_ levels promote synthesis and accumulation of SFAs in green algae and can lead to the desaturation of pre-existing fatty acids [63]. SFAs were also found to increase proportionally under elevated *p*CO_2_ conditions in diatoms and this effect was magnified in grazing copepods [31]. Our results on a predatory whelk are inconsistent with this, as we found a significant decrease in the relative proportion of SFAs under future CO_2_ acidification conditions. However, the whelks were fed fresh oysters that were held under current ocean conditions, and thus our study does not account for potential trophic transfer of altered lipid compositions. The integration species interactions into future studies on temperature and *p*CO_2_-induced biochemical changes could provide a more realistic assessment of species vulnerabilities, based on functional networks from the molecular to ecosystem scale [64].

### 2.3. Impacts of Ocean Climate Change on Fatty Acid Composition

In the present study, we have identified 21 fatty acids in *D. orbita* lipid extracts (Table 2, Figure S1), with carbon atoms ranging from 14 to 22, including saturated, monoenoic, polyenoic and cyclopropane-containing fatty acids (CFA) (e.g., Figure S2). The biophysical properties of these cyclic fatty acids are similar to unsaturated fatty acids, hence they are usually considered as part of the unsaturated fatty acid component [65]. Several studies have confirmed the presence of CFAs in marine organisms, such as the Okinawan ascidian *Diplosoma* sp. [66] and the Caribbean sponge *P. suberitoides* [67]. CFAs have also been previously reported in freshwater molluscs, specifically the prosobranch gastropods Viviparus (*Bellayma*) *bengalensis* and *Pila globosa* [68]. CFAs are common in bacteria and function to increase cellular membrane stability when bacteria are exposed to low pH [69]. Consistent with this we observed a slight increase in 2-octylcyclo-propanedecanoic methyl ester in whelks held under future CO_2_ conditions (Table 2). The relative proportion of CFAs in *D. orbita* also increased at elevated water temperatures, which may be due to the post-synthesis modification of unsaturated fatty acids [70].

**Figure 3:**
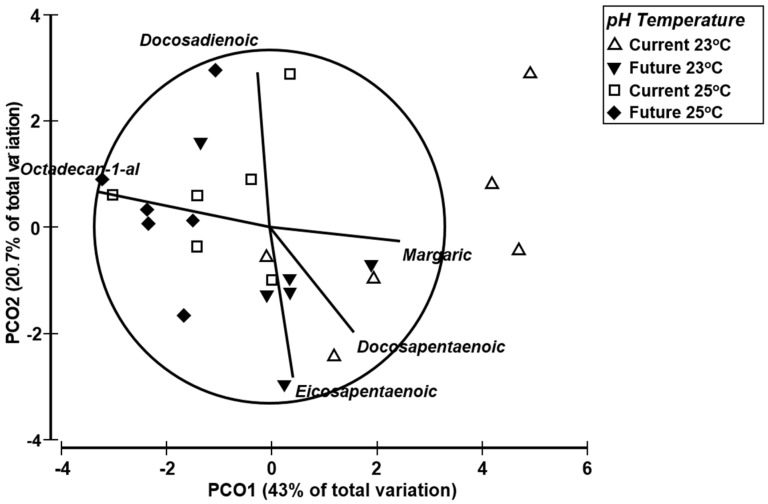
Principal coordinate ordination (PCO) of the lipophilic compound profile from *D. orbita* based on a Euclidian distance similarity matrix of the percent composition data with vector overlay from Spearman rank correlation of 0.6.

A series of dimethyl acetals of aliphatic aldehydes were also identified in the lipid composition of *D. orbita* (Table 2, Figure S3), based on the common fragment ion *m*/*z* 75, representing the McLafferty rearrangement ion (CH_3_O)_2_CH^−^ [71]. The detection of these dimethyl acetals of aldehydes in *D. orbita* tissue is consistent with the presence of animal cell plasmalogens, which are glycerolphospholipids with a vinyl ether linkage at the sn-1 position and an ester linkage at the sn-2 position [72]. The aldehydes are generated when the vinyl ether bond is broken, then immediately converted to dimethyl acetals during acidic transesterification in BF_3_ methanol [71]. Plasmalogens play many important physiological roles in animals and have been proposed to provide a sink for polyunsaturated fatty acids in some tissues [72]. In a study on the mitochondrial fraction of the marine bivalve *Arctica islandica*, the abundance of plasmalogens (evaluated on the basis of dimethyl acetal content) was found to slightly increase as a result of warmer temperature [73]. This is in agreement with our results. In *D. orbita*, the proportional increase of dimethyl acetals at elevated temperature coincides with the decrease of PUFA (Table 1, Figure 3). This may be because PUFAs are degraded by autoxidation chain reactions within the cell membranes, thereby releasing reactive fatty aldehydes [61,73]. Multivariate PERMANOVA revealed that there is a significant effect of both temperature and acidification on the overall fatty acid composition of *D. orbita* (Table 1, *p* < 0.05). However, there was no synergistic interaction between temperature and acidification (Table 1, *p* > 0.05). Principal coordinate ordination (PCO) with trajectory overlay was used to explore the differences in the lipid composition between temperature and acidification treatments (Figure 3). The whelks held at 23 °C and current *p*CO_2_ treatment are spread across the right hand side of the ordination plot, whereas the points representing whelks held at 25 °C and future *p*CO_2_ treatment are clustered mostly towards the left hand side (Figure 3). Vector overlay using Spearman rank correlation suggests that the whelks held at 25 °C in future *p*CO2 conditions are characterized by higher dimethyl acetal of octadecan-1-al, which could represent changes to the phospholipid cell membranes in whelks held under future stressful conditions. In comparison, the majority of whelks maintained at 23 °C in current *p*CO2 conditions are characterized by higher margaric acid and DPA (Figure 3, Table 2). Variation in the concentrations of docosadienoic acid and EPA along the *Y* axis explains much of the variation between replicate whelks held under the same treatment conditions (Figure 3).

Despite significant changes in the fatty acid composition of whelks held under future temperature and *p*CO_2_ conditions, the degree of impact on specific PUFAs was perhaps not as apparent as expected (Table 2). By comparison, a study on the gill of an eastern oyster *Crassostrea virginica* maintained at 25 °C showed a 14% increase in the amount of ARA compared to gills from the oysters kept at 12 °C. However, the magnitude of temperature change in this previous study on oysters was much greater (52% increase), in comparison to just 2 °C (8%) increase in our study, as this is more relevant to future ocean warming predictions. The detection of even relatively minor statistically significant changes to the fatty acid composition and glycerophospholipids of *D. orbita*, after just 35 days exposure to small predicted increases in water temperature, is of concern. These small changes in relative composition are magnified by the large decreases in total lipid content (Figure 1), with an average of 43% reduction in all lipids under future warming and *p*CO_2_ conditions, relative to current conditions. Long-term exposure and multi-generational studies are required to assess the potential for acclimatization and adaptation of the metabolic and biosynthetic capabilities of predatory marine whelks under future ocean conditions.

Furthermore, whilst this study has examined the impacts of ocean climate change stressors on whelk lipid storage and fatty acid composition, we have not accounted for bioaccumulated effects on dietary derived fatty acids likely to result from predator-prey interactions in natural ecosystems experiencing long term climate change. The combined effects of ocean warming and acidification have demonstrated effects on the biosynthesis of PUFAs in marine algae, with flow-on effects for herbivores [31] and ultimately this could further magnify the effects on higher order consumers. Therefore whilst the statistically significant changes in fatty acid proportions in *D. orbita*, along with the overall reduction in total lipid yield under future ocean conditions, can be considered a negative outcome from ocean warming and acidification, the effects may actually be under-estimated, with unknown consequences for the nutritional requirements and long-term survival of the species. Future mesocosm studies, involving a range of marine predator and prey interactions, could investigate the potential accumulation of biochemical changes in key primary metabolites under prolonged stressful conditions. This would help establish whether marine species at higher trophic levels are generally at greater risk of metabolic dysfunction under future ocean conditions and any consequent implications for the production of sustainable healthful seafood.

## 3. Experimental Section

### 3.1. Study Site and Experimental Design

To test the hypothesis that ocean warming and acidification will impact the fatty acid composition of *Dicathais orbita*, 144 whelks were subjected to 35 days under experimental conditions at the National Marine Science Centre (NMSC), Coffs Harbour, Australia (30°16′3.70″ S, 153°8′15.31″ E). The experiment utilized *D. orbita* (51–79 mm shell length) from rock platforms around Coffs Harbour and a two-factor factorial design with four treatments (mean ± SD): (1) current conditions, 22.9 ± 0.6 °C and 378.6 ± 35.6 ppm; (2) elevated temperatures, 25.2 ± 0.6 °C and 382.2 ± 35.5 ppm; (3) elevated *p*CO_2_ = 22.9 ± 0.5 °C and 749.9 ± 80.6 ppm; and (4) increased temperature 25.3 ± 0.6 °C and elevated *p*CO_2_ = 763.0 ± 104.6 ppm. Ambient water temperatures were based on data collated by Navy Meteorology and Oceanography indicating an average (±SD) sea surface temperature off the Coffs Harbour coast between September and November of approximately 21.3 ± 1.0 °C [74]. Ambient pH during the experimental period was estimated to be ~8.2 [75]. Future ocean conditions were based on the IPCC [76] trajectory for a drop in 0.3 pH (*i.e.*, increased *p*CO_2_ to 750–800 ppm) and a 3 °C rise in sea surface temperature by the year 2100 under climate change model RCP8.5.

To maintain experimental conditions, twelve 1100 L header tanks were filled with seawater pumped from the open ocean adjacent to the NMSC. This seawater was passed through a sand filter and 50 µm filters before being allowed to flow into the header tanks. The temperature of seawater in the header tanks was controlled by heater chiller units (Aquahort Ltd., Auckland, New Zealand). Seawater pH (*p*CO_2_) was manipulated by bubbling CO_2_-enriched air through experimental treatments after pre-mixing the gases using a gas mixer (PEGAS 4000MF, Columbus Instruments, Columbus, OH, USA). The water temperature, pH, conductivity and salinity were measured daily and total alkalinity was measured weekly through potentiometric titration using an automated titrator (888 Titrando, Metrohom, Riverview, FL, USA). The pH, alkalinity, temperature and salinity readings were used to calculate the partial pressure of CO_2_ using the CO2SYS program [77] with constants from Mehrbach *et al.* [78] as adjusted by [79].

Each header tank supplied temperature and *p*CO_2_ controlled water at 3 L/min^−1^ to a tray (860 mm × 650 mm × 96 mm) that housed four cages (305 mm × 360 mm × 90 mm) with three *D. orbita* in each cage. *D. orbita* were acclimated in experimental conditions for one week before feeding trials commenced. Whelks were fed Sydney rock oysters (*Saccostrea glomerata*), a common prey item in field conditions. The whelks in each cage were initially fed 4 small oysters ranging 30–50 mm, with new oysters of similar size added daily. After 35 days exposure to the experimental conditions, three male and three female whelks were sampled from each tank.

### 3.2. Extraction and Preparation of Fatty Acid Methyl Esters

To prepare the samples, soft tissue of the whelks was extracted by crushing the shell using a bench-top vice. Tissues from three replicate male whelks were pooled from each tank and similarly three replicate females were pooled to represent one replicate sample per tank (*n* = 3 tanks per treatment combination for each gender). The samples were prepared from the foot tissue (~1.0 gram) and were soaked in methanol:chloroform (2:1) for 1.5 h. The solvent extract was then filtered using Sigma-Aldrich Whatman filter paper 90 mm into a clean test tube. The tissue was soaked further in fresh solvent, decanted and replaced until a colourless solution was obtained. At least three washes were made to maximize lipid recovery. All the chloroform fractions from each sample were combined and dried using a rotary evaporator (Buchi Vacuum System) at 40 °C maintained at 337 mbar Hg. The samples were then transfered to a clean pre-weighed vial using sequential resuspension in minimum volumes of methanol:chloroform solution and were further concentrated under a stream of high purity (100%) nitrogen gas and then weighed and stored in a −20 °C freezer until utilized.

The above lipid extracts were derivatised to generate fatty acid methyl esters according to Kanthilatha *et al.* [80]. The dried lipid extracts were dissolved in 1.5 mL of 0.5 M saturated sodium hydroxide in methanol and then heated in a dri-block at 100 °C for 10 min. To completely methylate the extracts, 2 mL of boron trifluoride in methanol was added and then heated again for another 30 min. After cooling at room temperature, 1 mL of hexane was added to extract the fatty acid methyl esters (FAMEs). The tube was shaken vigorously for 30 s. Five milliliters of sodium chloride solution was added to aid in phase separation and shaken again for 5 s to separate the hydrophilic layer from the lipophilic layer. The FAMEs that were formed were recovered from the lower phase and collected in an autosampler vial ready for gas chromatography (GC) injection.

### 3.3. FAMEs and GC Analysis

The FAMEs samples were analysed using a GC (Agilent 6890N, Santa Clara, CA, USA) coupled with a flame ionisation detector (FID) with Agilent 6890 split/splitless injection and a fitted with BPX 70 capillary column (70% cyanopropyl polysilphenylene-siloxane, 50 m length, 0.22 mm internal diameter and 0.25 µm thickness). The FID was operated at 260 °C while the split injector was kept at 230 °C. The carrier gas was high-purity helium maintained with a linear flux of 1 mL/min. The GC oven was initially held at 100 °C for 5 min and then raised to 240 °C at a rate of 5 °C/min. One microliter of the sample extract was injected with a split ratio of 200:1 and a column flow of 1 mL/min.

FAMEs were identified by the peak, retention time and elution order and compared against the reference FAMEs standard test mix (SUPELCO 37-Component FAME Mix CRM47885, Bellefonte, PA, USA). Some samples were further analyzed using an Agilent gas chromatography-mass spectrometer (GC-MS) with an Agilent 5973 Mass Selective Detector to confirm the identity of the fatty acids. The mass spectra were recorded at 70 eV ionisation voltage over the mass range of 35 to 550 amu. Individual peaks were identified by comparison to library mass spectra (WILEY 275 and NIST98). To facilitate the identification of DPA, which was not in the test mix, a soft ionisation MS technique at 40 eV ionisation voltage was employed to ionise the lipid molecules in the *D. orbita* samples without causing extensive fragmentation (Figure S4). Spectrum was compared on the MS database, retention time and elution order from extensive literature search such as the American Oil Chemists’ Society. The relative composition of each identified fatty acid was done by peak integration from the GC and expressed for individual FAMEs as percentages of the total in each run.

### 3.4. Statistical Analyses

The data are expressed as means ± standard error. All statistical analyses were undertaken using PRIMER v 6 + PERMANOVA add-on. Initially three-factor PERMANOVAs (temperature, *p*CO_2_ and gender) were used to investigate the effects of temperature, *p*CO_2_-induced pH and snail gender. However, in all cases gender was not statistically significant and did not interact with the other factors (*p* > 0.05). Consequently, two-factor PERMANOVAs (temperature and *p*CO_2_) were used to investigate the effects of temperature and CO_2_-induced acidification. Univariate PERMANOVAs were used to compare the total yield of lipid extract, the percent SFA, MUFA and PUFA, as well as the percent of *n-*3 and *n-*6 fatty acids between each of the experimental conditions. Multivariate analyses were used to assess the total fatty acid composition (relative abundance standardized by the total). In all cases, Euclidean distance similarity matrices were created and PERMANOVAs were run using the full model and 9999 permutations of the data to determine overall differences between treatments followed by pairwise analyses on the interaction term when this was significant. Principal component ordination (PCO) was also undertaken to visually represent the patterns in the multivariate data (overall fatty acid composition standardized by total), with vector overlay using Spearman correlation >0.6. In all analyses, a statistically significant result was accepted for *p* < 0.05.

## 4. Conclusions

*D. orbita* has been found to contain a complex fatty acid composition with high PUFA levels and a good ratio of *n-*3 and *n-*6 fatty acids, which is typical of healthful seafood. Small temperature and *p*CO_2_ increases predicted with future ocean warming and acidification can, however, negatively impact the total yield of lipids and the overall fatty acid composition. Overall, our study indicated that ocean warming significantly decreases the proportion of PUFA and increases plasmalogen derived dimethyl acetals of aliphatic aldehydes, whilst elevated *p*CO_2_ decreases SFA and MUFA and alters the overall fatty acid composition. However, with the exception of total lipids, the percent changes in composition are relatively small and the *n-*3:*n-*6 ratio remains the same, suggesting minimal implications for human consumption of *D. orbita* as nutritional seafood. Nevertheless, there are possible implications for the long term viability of the species resulting from reduced lipid reserves and the potential for reduced bioaccumulation of lipids through the food web in predatory species.

## Supplementary Files

Supplementary File 1
