# Supplementary material for: Ocean Warming and CO2-Induced Acidification Impact the Lipid Content of a Marine Predatory Gastropod
Source: Mar Drugs. 2015 Sep 24;13(10):6019–37. doi: 10.3390/md13106019 (PMC4626677; doi:10.3390/md13106019)
Supplement: Supplementary File 1 [file marinedrugs-13-06019-s001.docx]

Supplementary Materials

Fatty Acid Composition of *D. Orbita* under Current Conditions

The common fatty acids in lipid extracts from *D. orbita* foot tissue were identified by comparison
to the FAME standards test mix (Figure S1). All peaks were further confirmed by using gas chromatography-mass spectrometry (GC-MS). In total, 21 fatty acid methyl esters were detected, including one cyclopropane containing fatty acid and one unknown derivative that showed no clear matches in the GC/MS databases (Figure S2).

A series of four dimethyl acetal derivatives of aldehydes were detected in the lipid extracts from our samples by FAMES and GC/MS (Figure S1 and S3). Dimethoxymethane ((CH_3_O)_2_CH^−^) has
a molecular mass of 75 and is a dominant fragment [1] ion associated with acetals, which are formed from aldehydes during the acidic methylation of plasmalogens [2]. Fragment ions of *m*/*z* 255, 269, 283, and 297 were recorded (Figure S3) corresponding to loss of a methoxyl ion from the dimethyl acetals of hexadecan-1-al, heptadecan-1-al, octadecan-1-al and nonadecan-1-al, respectively. Attempts to obtain the molecular ion of these dimethyl acetals using a soft-ionisation technique at 40 eV was not successful, as the associated peaks were no longer detectable by GC-MS after one week. This is consistent with the fact that hemiacetals formed from aldehydes are unstable at room temperature.

The identification of DPA (Figure S4) and other uncommon fatty acids which were not present in
the FAMES test mix was supported by GC-MS and by comparison of the full scan mass
spectrometric data collected from the literature [3,4]. The DPA isomer detected in *D. orbita* was confirmed as methyl 7,10,13,16,19-docosapentaenoate C22:5 (*n*-3) with omega ion at *m*/*z* 108 for the
*n*-3 double bond (Figure S4). However, the mass spectra for docosadienoic and eicosadienoic methyl esters were inconsistent with methylene-interrupted (*n*-6) fatty acids and showed that these are nonmethylene-interrupted dienoic fatty acids (NMID FA). Two homologous NMID FAs were identified, the C20:2 and C22:2. In the case of C22:2 NMID FA, the molecular ion is at *m*/*z* 350, and there are characteristic ions at *m*/*z* 338, 319, 301, 276, 238, 223, 207, 178. While the C20:2 NMID isomer has
a molecular ion at *m*/*z* 322 and characteristic ions at *m*/*z* 291, 248, 220, 207, 192, 178 and so forth for the loss of the carbon 14 with the associated methyl group. Both *de novo* synthesized C20:2 and C22:2 are the most frequently reported NMID FA and are irregularly distributed among different body
organs and tissues of molluscs such as gastropods and bivalves, usually as minor components. [1]. But in our results, C22:2 is among the three most abundant PUFA (~12%). Positions of double bonds at Δ5,11 and Δ5, 13 are typical for the C20:2 NMID FA, while for C22:2 NMID FA, Δ7, 13 and Δ7,15 are the commonly encountered structures [1].

| 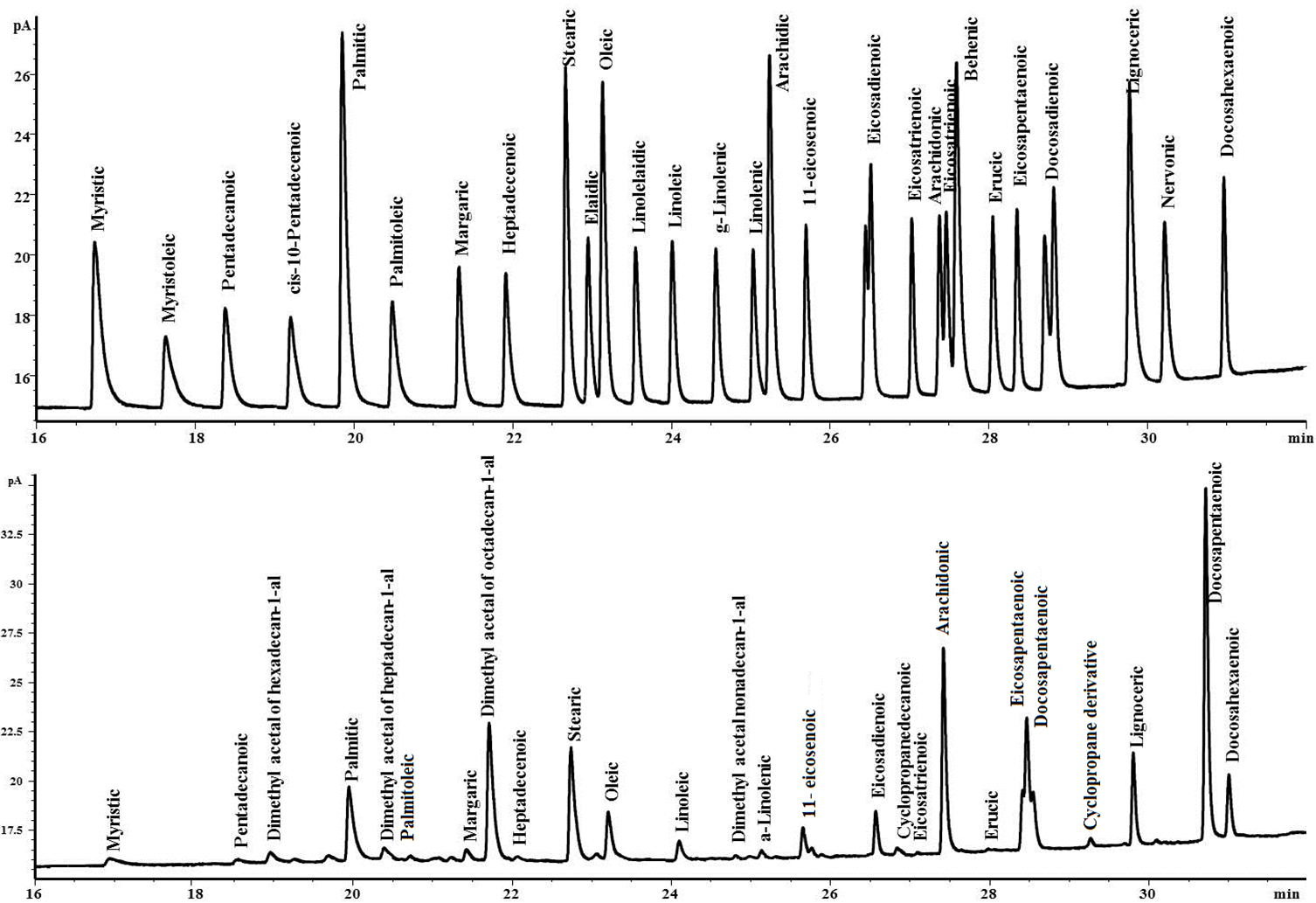 |
| --- |
| (**a**) |
| 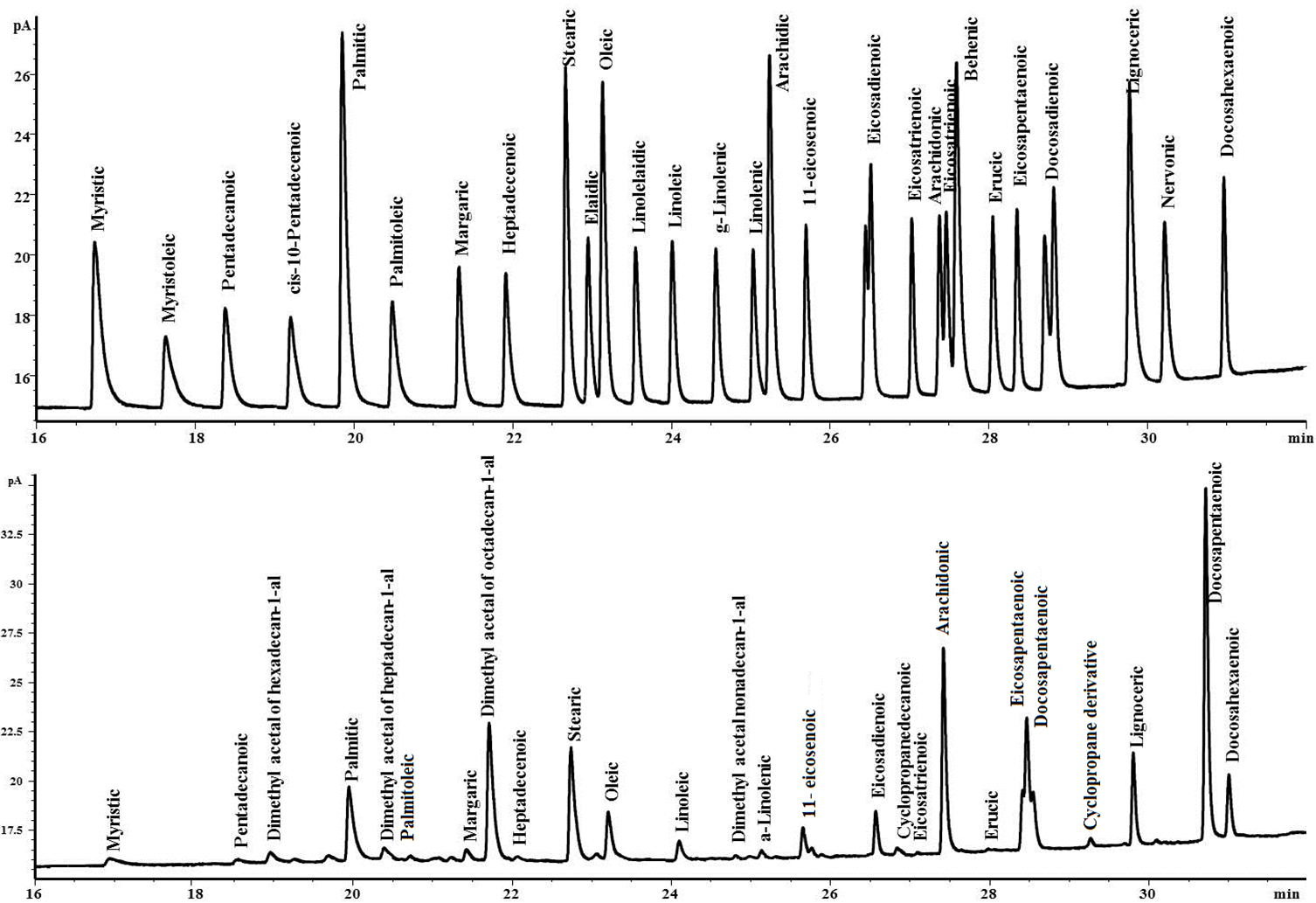 |
| (**b**) |

**Figure S1.** Gas chromatograms of fatty acid profiles from (**a**) FAMES test mix and (**b**) *Dicathais orbita* foot tissue.

| 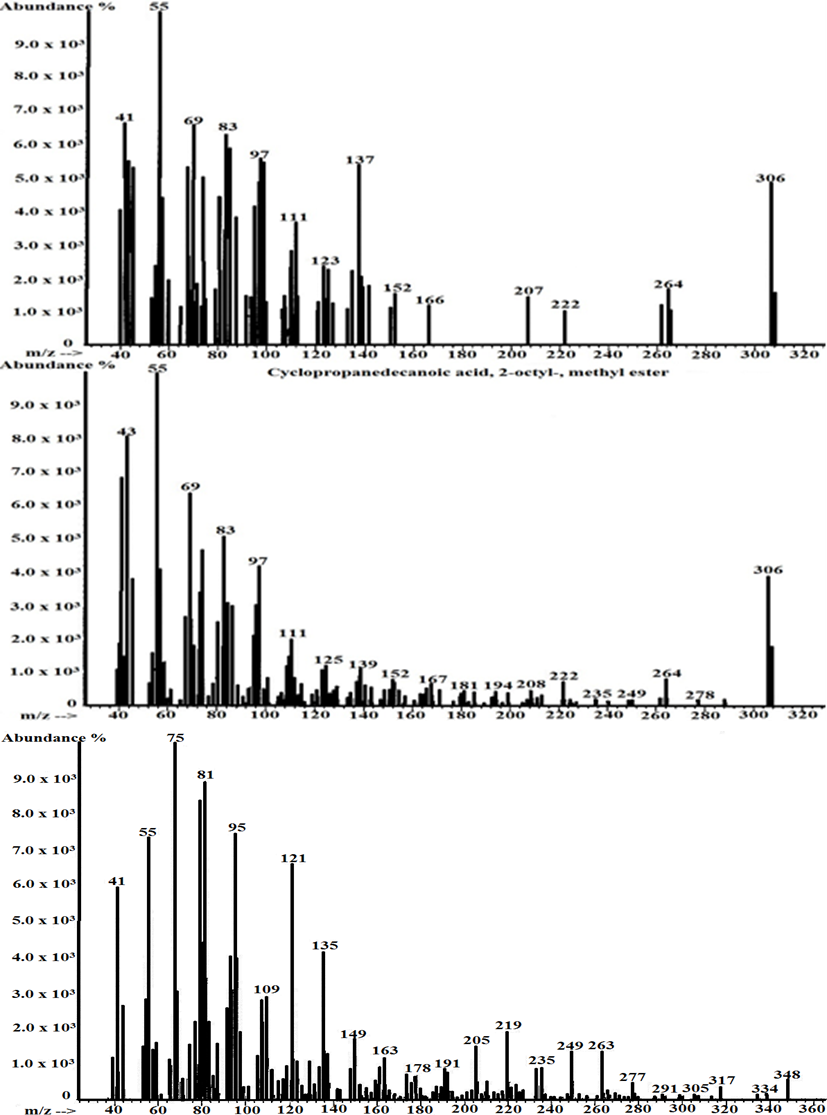 |
| --- |
| (**a**) |
| 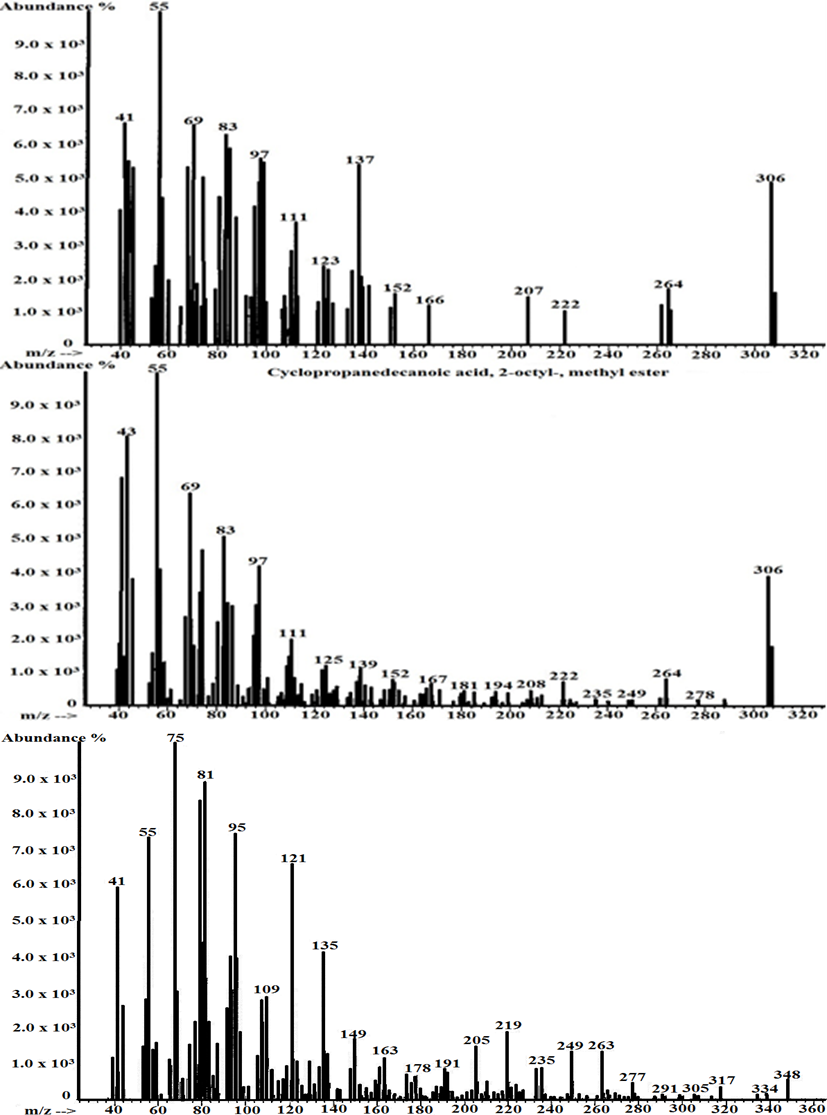 |
| (**b**) |
| 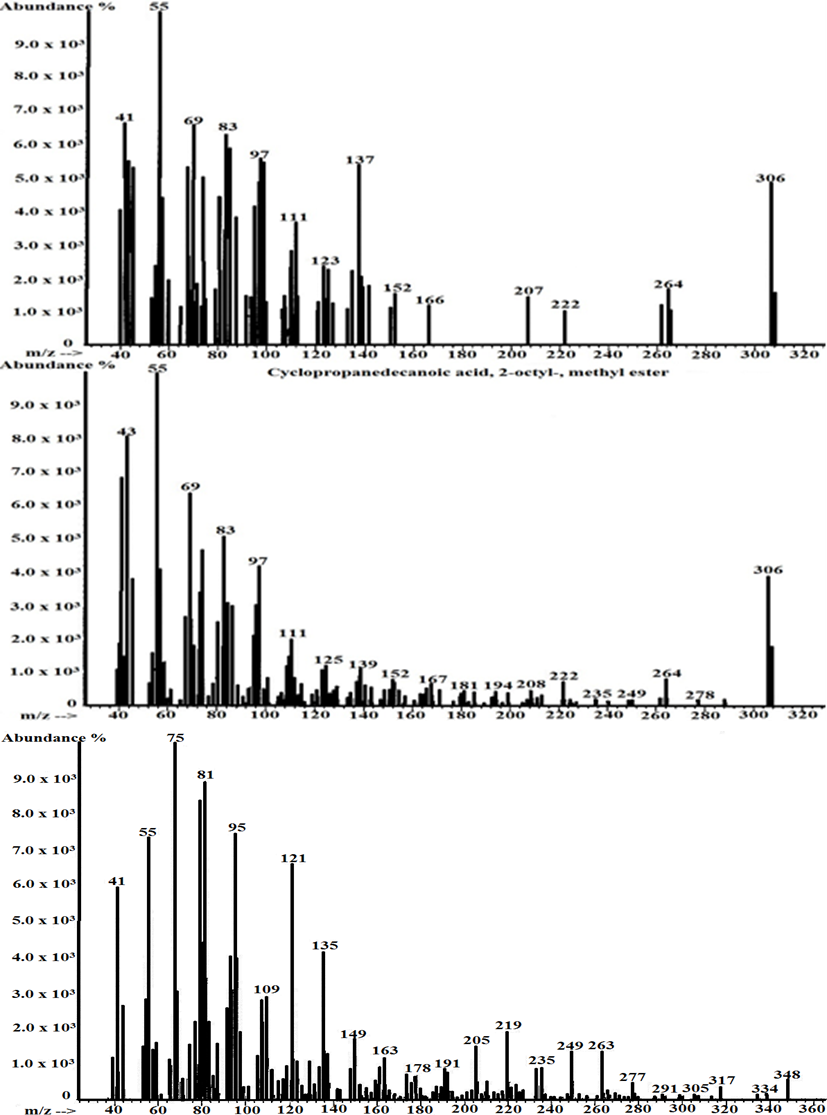 |
| (**c**) |

**Figure S2.** Mass spectra of cyclopropane-containing fatty acids from (**a**) *D. orbita* sample GC retention time, 26.8 min, showing a match to (**b**) 2-octylcyclopropanedecanoic acid methyl ester obtained from WILEY 275 and NIST98 database; (**c**) unknown fatty acid derivative from *D. orbita* sample with GC retention time 29.2 min.

| 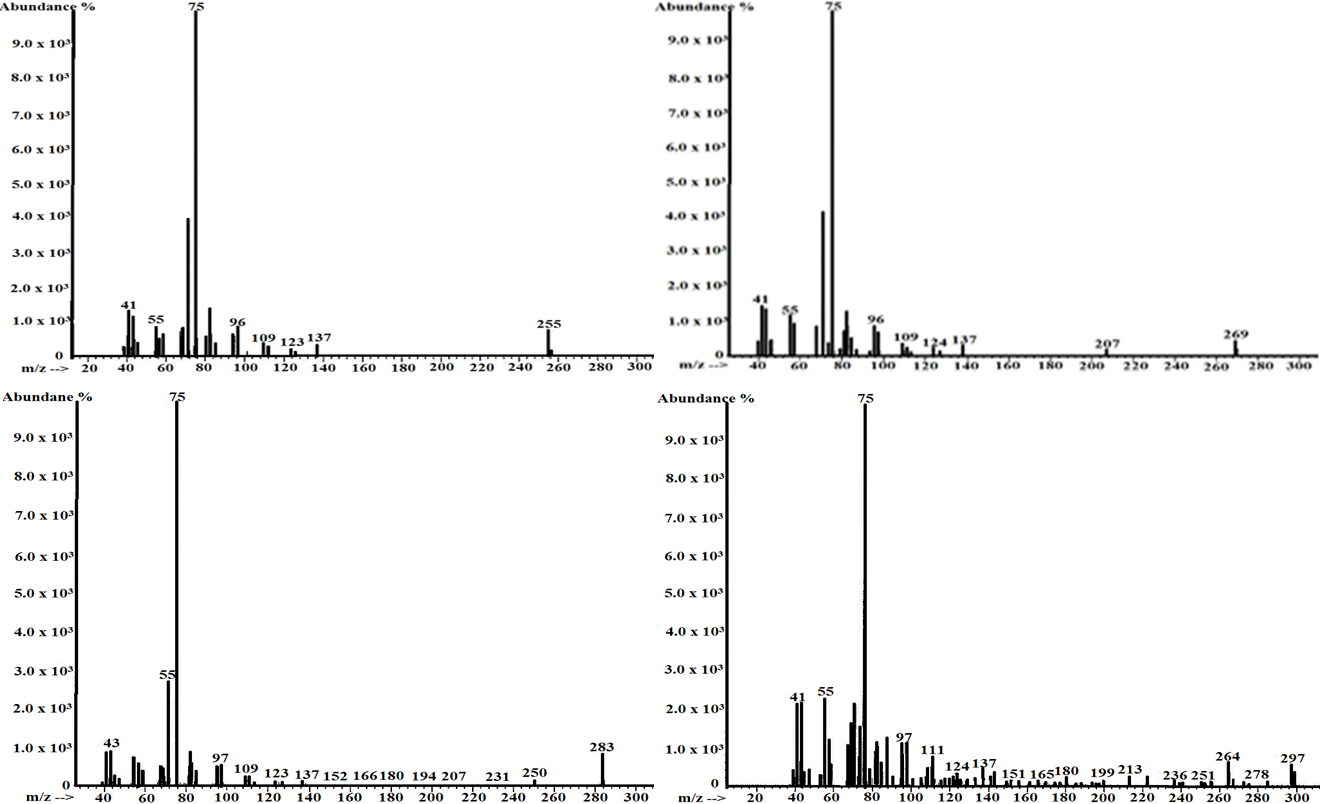 | 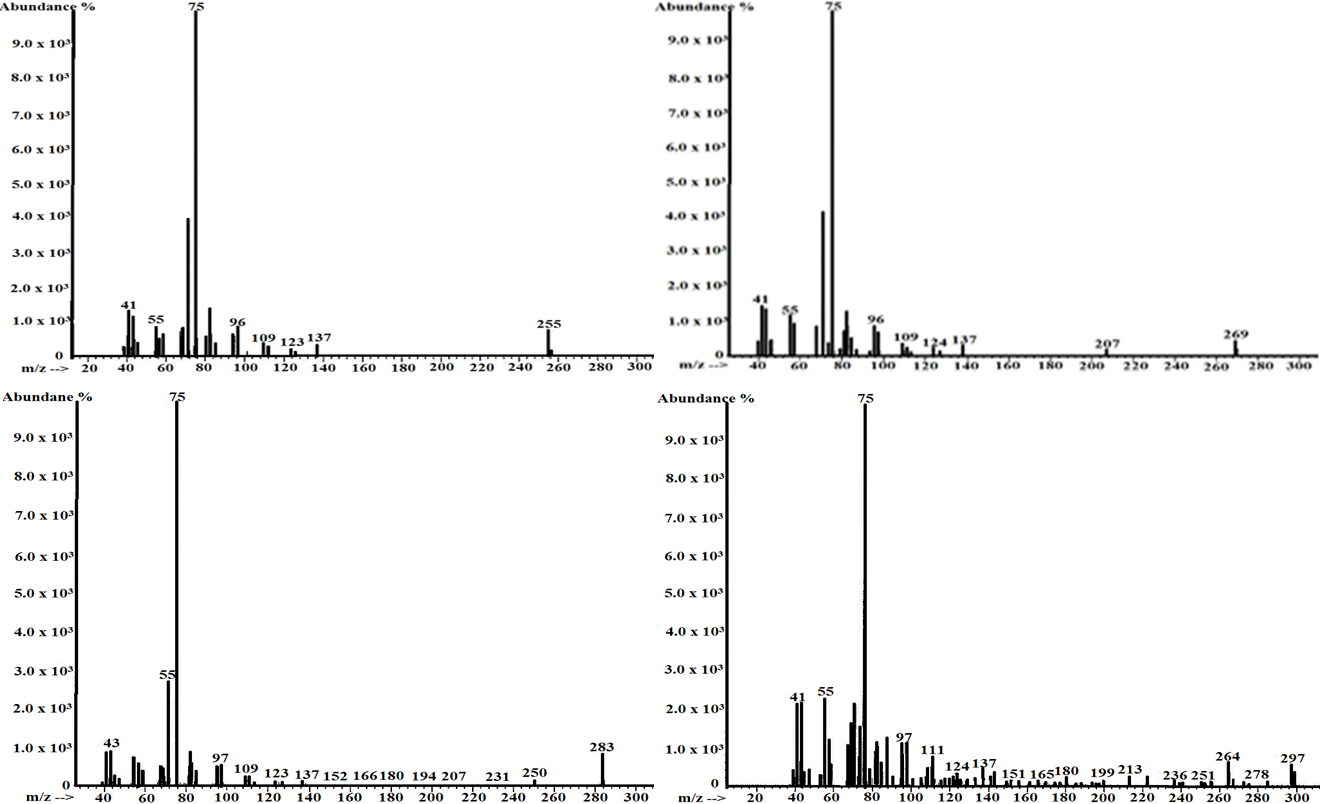 |
| --- | --- |
| (**a**) | (**b**) |
| 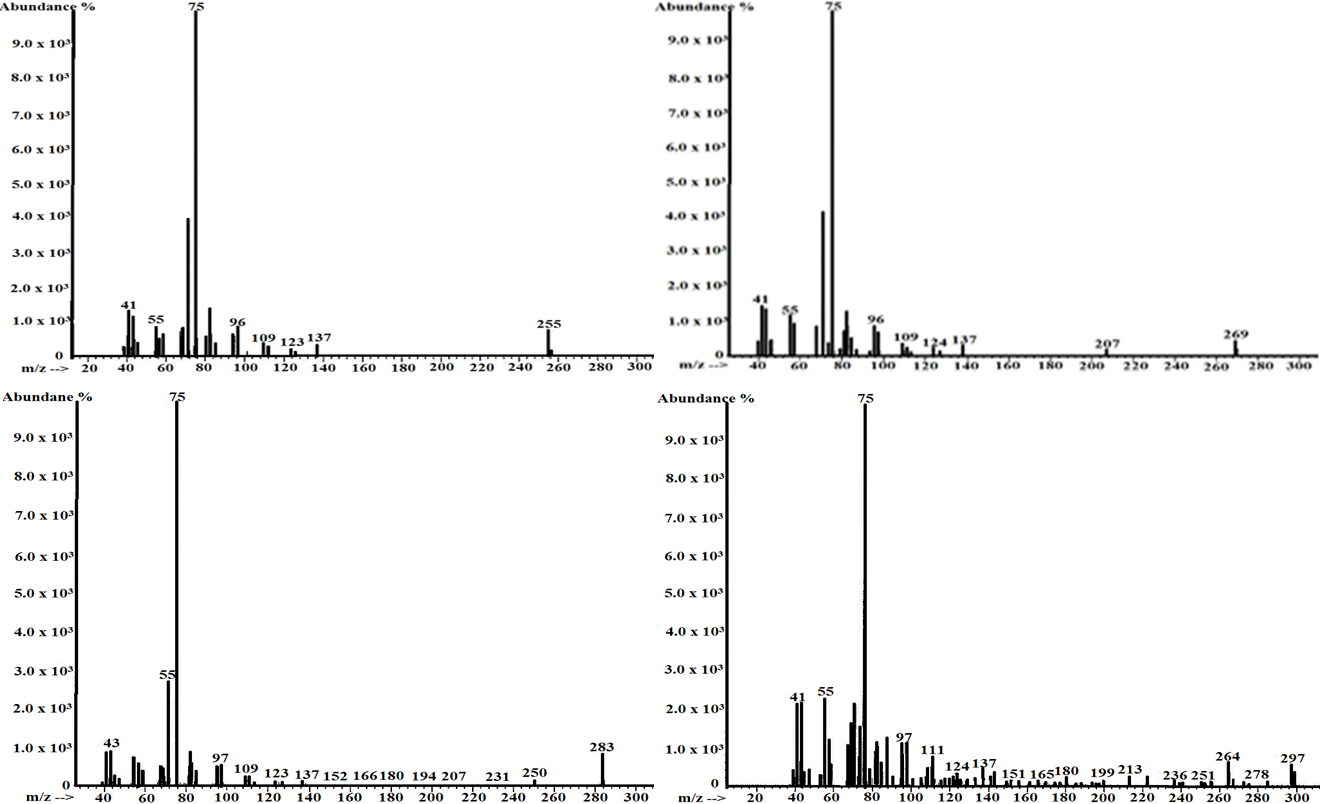 | 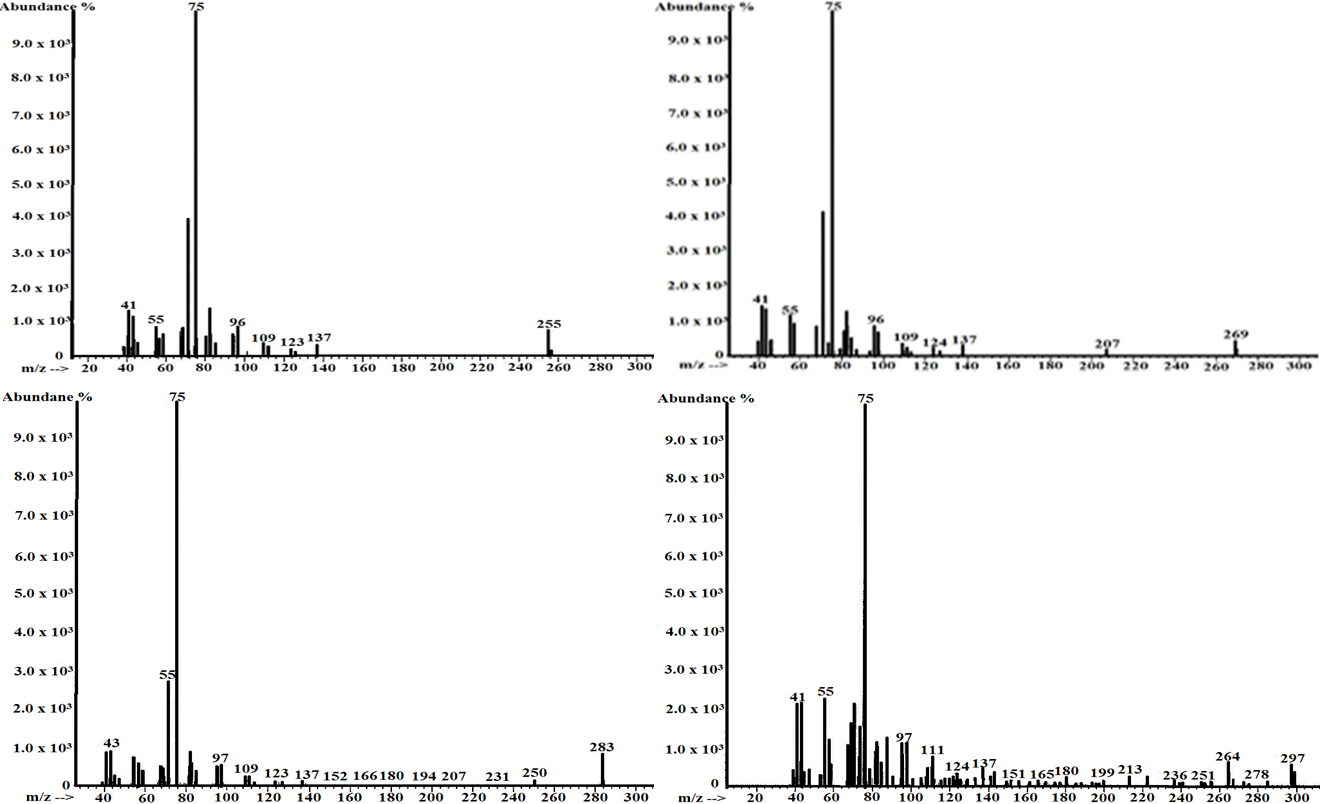 |
| (**c**) | (**d**) |

**Figure S3.** Mass spectra of a series of dimethyl acetals of aldehydes, showing *m*/*z* 75 as
a dominant fragment ion consistent with the McLafferty rearrangement ion ((CH_3_O)_2_CH^+^) and the loss of a methoxyl ion from the molecular ion in the high mass range; dimethyl acetals of (**a**) hexadecan-1-al, *m*/*z* 255 = M + (286) − OCH_3_, GC retention time = 18.8 min; (**b**) heptadecan-1-al, *m*/*z* 269 = M + (300) − OCH_3_, GC retention time = 20.2 min;
(**c**) octadecan-1-al, *m*/*z* 283 = M + (314) − OCH_3_, GC retention time = 21.7min and;
(**d**) nonadecan-1-al, *m*/*z* 297 = M + (328) − OCH_3_, GC retention time = 24.7 min.


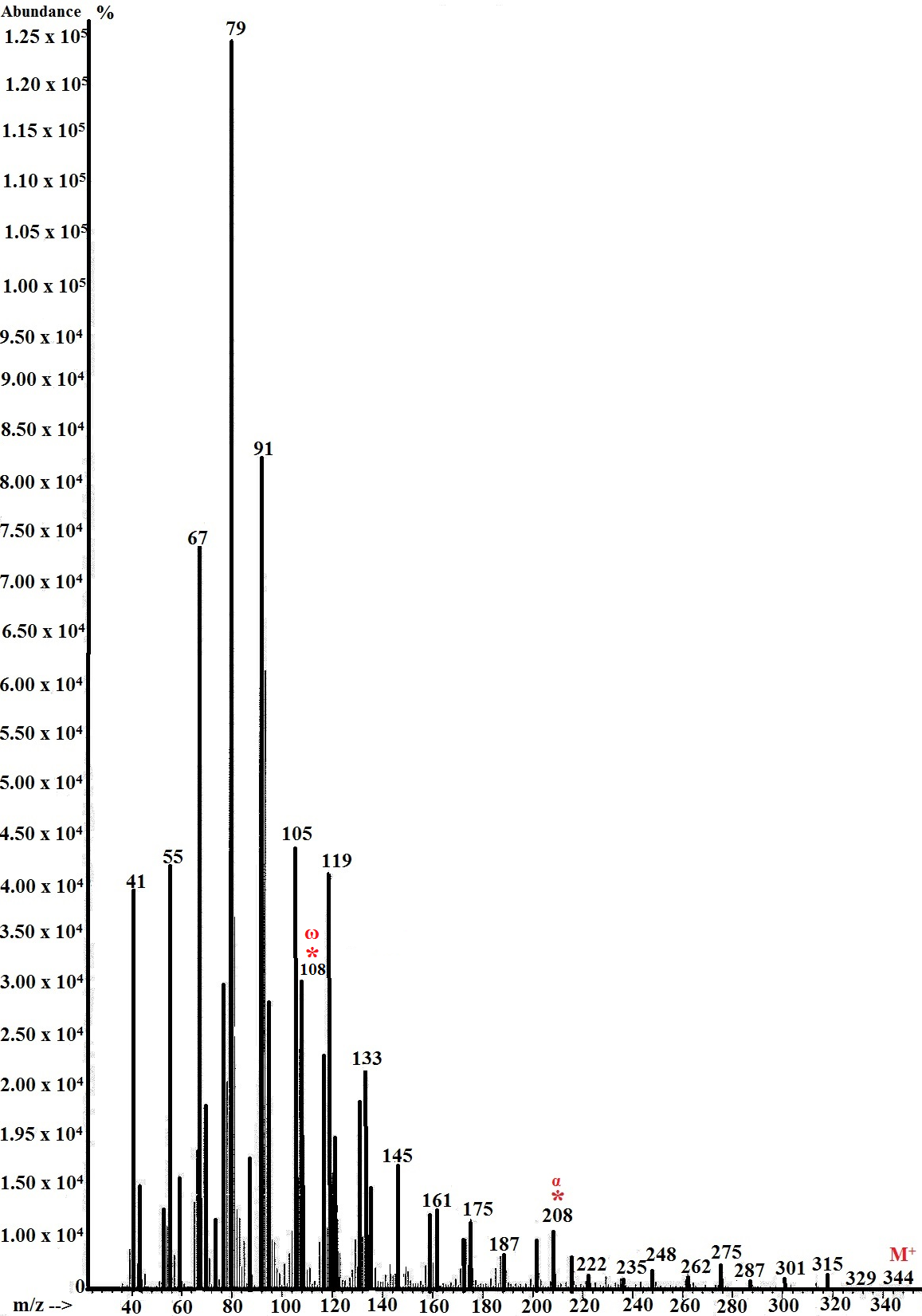


**Figure S4.** Mass spectrum of methyl 7,10,13,16,19-docosapentaenoate C22:5 (*n*-3) with omega ion at *m*/*z* 108 for an *n*-3 double bond.

References

1. Barnathan, G. Non-methylene-interrupted fatty acids from marine invertebrates: Occurrence, characterization and biological properties. *Biochimie* **2009**, *91*, 671–678.
2. Christie, W. Mass Spectra of Some Miscellaneous Lipophylic Components. Avaiable online: http://lipidlibrary.aocs.org/ms/ms21/index.htm (accessed on 12 June 2015).
3. Christie, W.W. Mass Spectra of Methyl Esters of Fatty Acids. Available online: http://lipidlibrary.
   aocs.org/ms/ms03e/index.htm (accessed on 31 May 2015).
4. Eder, K. Gas chromatographic analysis of fatty acid methyl esters. *J. Chromatogr. B* **1995**, *671*, 113–131.

© 2015 by the authors; licensee MDPI, Basel, Switzerland. This article is an open access article distributed under the terms and conditions of the Creative Commons Attribution license (http://creativecommons.org/licenses/by/4.0/).
